# Editorial: Tau Propagation Mechanisms: Cell Models, Animal Models, and Beyond

**DOI:** 10.3389/fnins.2020.00456

**Published:** 2020-05-19

**Authors:** Yumiko Motoi, Diane P. Hanger, Masato Hasegawa

**Affiliations:** ^1^Department of Diagnosis, Prevention and Treatment of Dementia, Juntendo University Graduate School of Medicine, Tokyo, Japan; ^2^Department of Basic and Clinical Neuroscience, Institute of Psychiatry, Psychology and Neuroscience, King's College London, London, United Kingdom; ^3^Department of Dementia and Higher Brain Function, Tokyo Metropolitan Institute of Medical Science, Tokyo, Japan

**Keywords:** tau, propagation, prion, aggregation, secretion

Tau is a major component of neurofibrillary tangles, one of the cardinal pathological features of Alzheimer's disease (AD) and tauopathies. A cross-sectional study showed that tangles typically spread from the medial temporal lobe to the limbic and association cortices in a stereotypical manner, paralleling symptomatic development in AD (Braak and Braak, 1991). Along with this classical observation, intracerebral injection of brain extracts containing aggregated tau or recombinant tau seeds, induces tauopathy in both tau transgenic and wild-type mice (Iba et al., 2013; Narasimhan et al., 2017). Tau aggregation can also be induced using tau seeds from cultured cells (Frost et al., 2009; Matsumoto et al., 2015). These phenomena strongly support the ability of tau protein to transfer from one cell to another as “prion-like” particles, similar to aggregated prion protein in Creutzfeldt-Jacob disease (Jucker and Walker, 2013). The prion-like transfer of misfolded tau aggregates involves secretion of proteins from donor neurons, uptake by recipient neurons, followed by the seeded assembly of misfolded host proteins in the recipient neurons. The molecular mechanisms underlying tau propagation are currently one of the most active areas of research in dementia.

This *Frontiers in Neuroscience Research Topic* comprises a highly informative compilation of original research and reviews covering multiple aspects of current research into tau propagation mechanisms. The topics include protein structure analysis, in which Cieplak describes mechanisms underlying tau fibrillization using a perturbational molecular orbital theory informed approach, with a focus on the electronic configuration and hyperconjugation of peptide amide bonds. Cieplak also references the recently discovered strain-specific differences in the structure of tau filaments revealed by cryo-electron microscopy (EM) (Fitzpatrick et al., 2017; Falcon et al., 2018, 2019). These studies report that, in three-repeat tau isoforms, only 2 of the 3 tau repeats are within the core of tau filaments isolated from AD and chronic traumatic encephalopathy (CTE) brain, but all 3 repeats are incorporated in the core of tau filaments in Pick's disease. Contact between tau protofilaments in Pick's disease occurs in the center of the third repeat; however, in AD and CTE, this contact is between the third and fourth repeats of tau. These differences are related to altered patterns of microtubule binding domain polarization brought about by protonation of His268 and His362, and suggest that one possible reason for strain-specific tau fibrillization may be differences in environmental pH. Recently, the structure of the four-repeat tauopathy, corticobasal degeneration (CBD), has been elucidated using cryo-EM (Arakhamia et al., 2020; Zhang et al., 2020). In CBD, three of the four repeats are incorporated within the core and contact between filaments occurs inside the fourth repeat in tau. This result would also be related to the same scenario.

Extracellular release of tau may be an active process that occurs in live neurons or passively by tau release from dead or dying cells (Pooler et al., 2013; Clavaguera et al., 2015). Tanaka et al. elegantly describe the biochemical differences between extracellular seed-competent tau and inert tau using biosensor cells expressing frontotemporal dementia-associated mutant P301S tau. Seed-competent tau in culture medium was enriched in a high molecular weight (>2,000 kDa) fraction, whereas the majority of soluble tau was enriched in low molecular weight fractions. Tanaka et al. also show that bafilomycin A1 or chloroquine, compounds that differentially impair lysosomal function, increase seed-competent tau. Pernègre et al. provide a comprehensive review of current knowledge of tau secretion pathways in relation to CSF-tau and suggest muscarinic receptors as a possible physiological target of extracellular secreted tau. Pernègre et al. also speculate that in pathological conditions, extracellular tau may interfere with synaptic function. Using antibody therapy, sequestration of secreted pathological tau in the extracellular space may prevent synaptic dysfunction. The review also noted that the amount of tau in the CSF differs between tauopathies. In AD, where the progression of the disease may be slow, increased tau in CSF is observed, whereas in progressive supranuclear palsy (PSP) and CBD, in which disease progression may be more rapid, either no change or a small decrease in CSF tau was noted. Pernègre et al. speculate that this observation might reflect lower tau secretion and higher propagation capacity of PSP and CBD tau strains, phenomena that could be related to the disease-specific differences in tau structure observed by cryo-EM (Fitzpatrick et al., 2017; Falcon et al., 2018, 2019; Arakhamia et al., 2020; Zhang et al., 2020).

Tau is mainly expressed in neurons, and glial cells generally do not contain appreciable amounts of endogenous tau. Using Cy5-labeled tau and rat primary cultures, Perea et al. found that monomeric tau is taken up by astrocytes. Heparan sulfate proteoglycans do not appear to be involved in this process, unlike the uptake of aggregated tau into various cell lines. Neuropathologic studies have shown various types of tau-positive astrocytes in conditions including aging-related tau astrogliopathy, globular glial tauopathy, as well as in neuropathological entities such as thorn-shaped astrocytes, tufted-astrocytes, and astrocytic plaques, that characterize distinct tauopathies. Thus, tau internalization by astrocytes could contribute to the development of these pathologies and/or to aging of astrocytes.

Takeda reviews recent literature on the propagation of tau *in vitro*, in animal models, and in human pathological conditions. Noting that many unresolved questions remain regarding the tau propagation hypothesis, Takeda highlights that there is no clear evidence of biological similarity between the structure of tau aggregates that form in cultured mouse neurons and in human AD brain. However, Takeda points out that identifying the most important tau epitope(s) will be critical for the development of therapies targeting tau propagation in tauopathies as this may be mediated by extracellular tau.

Overall, the articles within this *Frontiers in Neuroscience Research Topic* discuss recent and interesting findings relating to tau propagation in the tauopathies. We anticipate that further research on this topic will expand our knowledge on tau propagation and aggregation and provide important insight into the development of therapeutic approaches targeting tau propagation.

## Author Contributions

YM, DH, and MH contributed to the manuscript writing. DH contributed to review the articles of molecular biology. MH contributed to review the manuscripts of protein chemistry.

## Conflict of Interest

The authors declare that the research was conducted in the absence of any commercial or financial relationships that could be construed as a potential conflict of interest.
